# Internet Addiction and Related Clinical Problems: A Study on Italian Young Adults

**DOI:** 10.3389/fpsyg.2020.571638

**Published:** 2020-11-10

**Authors:** Lorenzo Zamboni, Igor Portoghese, Alessio Congiu, Silvia Carli, Ruggero Munari, Angela Federico, Francesco Centoni, Adelelmo Lodi Rizzini, Fabio Lugoboni

**Affiliations:** ^1^Department of Neurosciences, University of Verona, Verona, Italy; ^2^Unit of Addiction Medicine, Department of Internal Medicine, Integrated University Hospital of Verona, Policlinico “G.B. Rossi”, Verona, Italy; ^3^Department of Medical Sciences and Public Health, University of Cagliari, Cagliari, Italy; ^4^Amici C.A.S.A. San Simone No Profit Association, Mantova, Italy

**Keywords:** somatization, internet addiction, adolescent, moderation, obssessive-compulsive disorder

## Abstract

The considerable prominence of internet addiction (IA) in adolescence is at least partly explained by the limited knowledge thus far available on this complex phenomenon. In discussing IA, it is necessary to be aware that this is a construct for which there is still no clear definition in the literature. Nonetheless, its important clinical implications, as emerging in recent years, justify the lively interest of researchers in this new form of behavioral addiction. Over the years, studies have associated IA with numerous clinical problems. However, fewer studies have investigated what factors might mediate the relationship between IA and the different problems associated with it. Ours is one such study. The Italian version of the SCL-90 and the IAT were administered to a sample of almost 800 adolescents aged between 16 and 22 years. We found the presence of a significant association between IA and two variables: somatization (β = 7.80; *p* < 0.001) and obsessive-compulsive symptoms (β = 2.18; *p* < 0.05). In line with our hypothesis, the results showed that somatization predicted the relationship between obsessive-compulsive symptoms and IA (β = −2.75; *t* = −3.55; *p* < 0.001), explaining 24.5% of its variance (Δ*R*^2^ = 1.2%; *F* = 12.78; *p* < 0.01). In addition, simple slopes analyses revealed that, on reaching clinical significance (+1 SD), somatization showed higher moderation effects in the relationship between obsessive-compulsive symptoms and IA (β = 6.13; *t* = 7.83; *p* < 0.001). These results appear to be of great interest due to the absence of similar evidence in the literature, and may open the way for further research in the IA field. Although the absence of studies in the literature does not allow us to offer an exhaustive explanation of these results, our study supports current addiction theories which emphasize the important function performed by the enteroceptive system, alongside the more cited reflexive and impulsive systems.

## Introduction

*Internet addiction* (IA), also referred to as *problematic*, *pathological*, or *compulsive Internet use*, is a controversial concept in the research field. The frequent use of different terms to describe this new phenomenon, linked to the advent and growth of the Internet, leads to confusion over what it really consists of Tereshchenko and Kasparov (2019).

Although researchers have yet to find a common definition of IA, it can be considered a “*non-chemical, behavioral addiction, which involves human-machine interaction*” (Griffiths, 2000). Useful clinical criteria were proposed by Block (2008), who associates IA with (a) increased feelings of anger, anxiety or sadness when the Internet is not accessible (craving); (b) the need to spend more hours on Internet devices in order to feel pleasure or cope with dysregulation of mood (tolerance); (c) poor school performance or vocational achievement; and (d) isolation or social withdrawal.

One aspect that researchers agree on is the importance of IA prevention in children and adolescents (Lan and Lee, 2013). As with other forms of addiction, younger people are at greater risk of the negative effects of out-of-control Internet use (Ko et al., 2008). In adolescence, distress is expressed in the form of behavioral agitation, somatic symptoms, boredom and an inclination to act (Carlson, 2000), all modalities that facilitate the development of a coping strategy based on compulsive Internet use. This is a problem, given that 80% of adolescents use tablets or smartphones (Fox and Duggan, 2013), whereas general population prevalence rates range from 0.8% (Italy) to 26.5% (Hong Kong) (Kuss et al., 2014).

We currently know that IA is associated with symptoms of ADHD in teens (Yoo et al., 2004), pathological gambling (Phillips et al., 2012), depression (Andreou and Svoli, 2013; Ho et al., 2014), anxiety (Griffiths and Meredith, 2009; Zboralski et al., 2009), social phobia (Carli et al., 2013; Gonzalez-Bueso et al., 2018), experiential avoidance (Hayes et al., 1996; García-Oliva and Piqueras, 2016), obsessive-compulsive disorder (OCD) (Jang et al., 2008; Cecilia et al., 2013), eating disorders (Shapira et al., 2003; Bernardi and Pallanti, 2009), and sleep disorders (Nuutinen et al., 2014; Tamura et al., 2017), as well as with relational conflicts (Gundogar et al., 2012), aggression (Cecilia et al., 2013), self-destructive behaviors (Sasmaz et al., 2014), suicidal behaviors (Durkee et al., 2016), physical health problems (Sung et al., 2013), and chronic pain syndrome (Wei et al., 2012). However, little is known about the factors potentially implicated in the etiopathogenesis of IA (Tereshchenko and Kasparov, 2019).

Many of the most common symptoms of addiction and OCD are similar to each other, to the point that some authors define IA as compulsive computer use (Kuss et al., 2014). However, there are also significant differences between the two sets of psychopathological symptoms. The obsessive-compulsive symptoms that characterize OCD can be described as recurring and persistent inappropriate thoughts (obsessions) that lead the individual to implement behaviors (compulsions) aimed at reducing the intensity of the distress deriving from these obsessive thoughts (American Psychiatric Association [APA], 2013). Instead, the obsessive-compulsive symptoms reported in the context of addiction can also derive from positive thoughts about the object of the addiction, which drive the individual to seek and, in this case, engage in the activity in order to obtain gratification (Robbins and Clark, 2015).

In this framework, the obsessive-compulsive component of IA can be considered in terms of (a) recurrent positive and negative thoughts (obsessions), associated, respectively, with the memory of the enjoyable experience of using the Internet, and with craving or withdrawal syndrome; and (b) instrumental behaviors (compulsion) geared toward seeking the former (positive reward) or reducing the discomfort associated with the latter (negative reward).

Adolescents with IA can be expected to display: (a) a lower ability to use reflexivity to manage their internal states; and (b) a greater propensity for impulsive behaviors to manage these states. This is the hypothesis recently proposed by Wei et al. (2017) to explain internet gaming disorder (IGD), a form of IA. However, alongside the presence of a hypoactive reflective system and an overactive impulsive system, these authors also hypothesize a dysregulation of the interoceptive awareness system, and suggest that this dysregulation increases the incentive salience of Internet use, as well as the feeling of craving deriving from its compulsive use (Wei et al., 2017). This thesis could explain the relationship commonly observed between compulsive use of the Internet and somatization (Yang et al., 2005).

Somatization is defined as the “*unconscious process of expressing psychological distress in the form of physical symptoms*” (Nakkas et al., 2019), and it is commonly found among adolescents with IA. It is estimated that 9% of Internet-addicted adolescents display somatization (Yang, 2001), reported in the literature to consist of somatic symptoms (Potembska et al., 2019), chronic pain (Wei et al., 2012; Fava et al., 2019), physical health problems (Sung et al., 2013), and sleep disorders (Tamura et al., 2017). Moreover, in late adolescence, the presence of somatization has been positively associated with the intensity of specific forms of IA, such as IGD (Cerniglia et al., 2019). One study showed that higher somatization and interpersonal sensitivity scores predict problematic smartphone use (Fırat et al., 2018). Ballespi et al. (2019), illustrate that inability to mentalize is associated with a higher frequency of somatic complaints.

Although the involvement of somatization in the etiopathogenesis of IA is not yet clear, models recently advanced to explain the development of addiction assign it a primary role. In the triadic neurocognitive model of addiction (Noël et al., 2013), for example, perception of the somatic state of the organism, governed by the insular cortex, is considered a factor that mediates the development of addiction. In fact, in the absence of cognitive processing of the bottom-up somatic signals mediated by this cerebral structure, the main symptoms of addiction suddenly disappear.

These data were recently confirmed by Naqvi et al. (2007), who showed that absence of the somatic symptoms typical of craving and physical abstinence, induced by ischemic damage to the insula, allowed heavy smokers to give up smoking.

Somatization has been reported in association with IA in a college student population (Alavi et al., 2011), and it has also been identified among the causal factors and predictors of IA among first-year college students (Yao et al., 2013). Indeed, this latter study confirmed that students with somatization seem to have a greater tendency to develop IA. In addition, a study by Biby (1998) showed that higher somatization scores are linked to higher obsessive-compulsive tendency scores. Therefore, if a key role of somatic symptoms in modulating the activity of the reflexive and impulsive systems can be taken to explain the development of IA, it seems possible to hypothesize that the presence of obsessive-compulsive symptoms, commonly found in adolescents with IA (Yen et al., 2008), may also be linked to the presence of somatization. In this sense, an additional hypothesis is that higher somatization in adolescents might exacerbate the effect of obsessive-compulsive symptoms on IA. Surprisingly, this hypothesis has not been investigated in the literature to date, although contemporary etiopathogenetic models suggest the importance of bottom-up somatic signals in addiction disorders (Verdejo-García and Bechara, 2009). In fact, somatic symptoms may be linked to the presence of the same top-down processing of body signals related to craving or abstinence. According to the above hypothesis, these symptoms may upset the activity of the cognitive system, shifting it away from inhibitory control of Internet use, implemented by the reflexive system, toward compulsive behaviors, driven by the impulsive system (Wei et al., 2017). In this way, high levels of somatization could both promote the development of IA and reinforce the relationship between obsessive-compulsive symptoms and IA.

In conclusion, our hypothesis is that somatization moderates the positive relationship between OC symptoms and IA. Specifically, the higher the level of somatization, the stronger the relationship.

## Materials and Methods

### Participants

Participants were recruited from schools in the north of Italy. The study was presented during the participants’ classes. Students were invited to take part in a research study that aimed to investigate: drug use/abuse, gambling problems, alcohol use/abuse, mood. Students who provided informed consent were given two self-report instruments. All the participants were free to stop filling in the questionnaires at any time. Underage subjects needed parental permission to participate in this study.

The participants (57.7% females) ranged in age from 16 to 22 years (mean age 17.52 ± 1.15). All were third (35%), fourth (37%), or fifth grade (28%) Italian secondary school students.

### Instruments

#### Symptom Checklist 90—Revised (SCL-90-R; Derogatis, 1994)

The Somatization (SOM;12 items) and Obsessive-Compulsive (OC; 10 items) subscales of the Italian version of the SCL-90-R were used. The participants used a five-point Likert scale, ranging from 0 (Not at all) to 4 (Extremely), to rate the extent to which they had experienced the listed symptoms during the past week. Cronbach’s alpha was 0.86 for SOM, and 0.82 for OC.

#### Internet Addiction Test (IAT; Young, 1998)

This is a 20-item questionnaire on which respondents are asked to rate, on a five-point Likert scale, items investigating the degree to which their Internet use affects their daily routine, social life, productivity, sleeping patterns, and feelings. The minimum score is 20, and the maximum is 100; the higher the score, the greater the problems caused by Internet use. Young suggests that a score of 20–39 points is that of an average on-line user who has complete control over his/her Internet use; a score of 40–69 indicates frequent problems due to Internet use; and a score of 70–100 means that the individual’s Internet use is causing significant problems. Cronbach’s alpha was 0.88.

### Socio-Demographics

The participants reported their age, gender, school and grade. In order to maintain privacy, no other personal information was requested.

### Control Variables

The use of illicit drugs and gambling behavior were introduced as control variables. Specifically, the participants answered questions on their habits regarding any use of illicit drugs (cannabis, cocaine, heroin), alcohol consumption, and gambling activities, such as scratch cards, lottery tickets, football pools, new slot machines (VLTs) and video poker, betting on sporting or other events, poker and other card games.

### Statistical Analyses

All the analyses were carried out using IBM SPSS Statistics 26.0 and AMOS (Arbuckle, 2012). A series of confirmatory factor analyses (CFAs) was conducted to establish the discriminant validity of the scales. A full measurement model was initially tested, comparing it to a one-factor structure (in which all the items loaded into a common factor). The model fit was tested by using the comparative fit index (CFI), the incremental fit index (IFI), and the root-mean-square error of approximation (RMSEA). According to Kline (2008) and Byrne (2016), the CFI and IFI values should have a cutoff value of ≥0.90, and the RMSEA a value of ≤ 0.08 to indicate a good fit of the model. Internal consistency of the constructs was evaluated using Cronbach’s alpha (α).

We tested the effects of somatization symptoms, obsessive-compulsive symptoms, and their interaction on IA by using the SPSS version of Hayes’s (2017) bootstrap-based PROCESS macro (Hayes, 2012, 2013; Model 1). All predictors were mean-centered prior to computing the interaction term and simple slopes were calculated at ± 1 SD. Age, sex, type of school, grade, use of illicit drugs, and gambling behaviors were included as covariates. To account for non-normality, analyses were performed with bootstrapping with 5,000 resamples.

## Results

### Preliminary Analyses

Table 1 shows the means, standard deviations and internal consistencies obtained for each scale, and the correlations between the measures used in the current study.

**TABLE 1:** Descriptives of study variables (*n* = 796).

	Mean	*SD*	1	2	3
Internet addiction test	38.11	10.96	(0.88)		
Somatization subscale of the SCL-90-R	0.73	0.61	0.29***	(0.86)	
Obsessive-compulsive subscale of the SCL-90-R	0.93	0.62	0.44***	0.57***	(0.82)

***p < 0.001.

#### Measurement Model

Prior to testing our hypothesis, we used CFAs to examine the convergent and discriminant validity of our study variables. The data were found to fit the measurement model: χ^2^(811) = 1150.99, *p* < 0.001, CFI = 0.90, TLI = 0.90, RMSEA = 0.035. All items loaded significantly on the intended latent factors.

#### Moderation Analysis

It was hypothesized that obsessive-compulsive symptoms would predict IA, depending on the somatization symptoms (moderation hypothesis). Regression analyses (Table 2) conducted with the PROCESS macro (Model 1; Hayes, 2012) showed that obsessive-compulsive symptoms (β = 7.80, *p* < 0.001) and somatization symptoms (β = 2.18, *p* < 0.05) were related to IA after controlling for age, sex, grade, school, illicit drug use, and gambling behaviors. The moderation effect was significant *t*(788) = −3.55; *p* < 0.001 (β = −2.75, *SE* = 0.77, CI −4.27 to −1.2) and accounted for a significant portion of variance of IA [Δ*R*^2^ = 1.2%; *F*_(__788__)_ = 12.78 *p* < 0.01]. In this sense, increasing obsessive-compulsive symptoms predicted increased IA, but this effect was greatest at higher levels of somatization symptoms. The final model accounted for a total of 24.5% of the variance in IA.

**TABLE 2:** Results of the moderation analysis.

	β	*SE*	*t*	*p*	95% CI	
Intercept	44.6	5.40	8.27	< 0.001	34.01	55.19
Sex	–2.58	0.74	–3.49	< 0.001	–4.03	–1.13
Age	0.39	0.35	1.12	ns	–0.29	1.08
Grade	–2.68	0.51	–5.29	< 0.001	–3.67	–1.69
School	–0.64	0.51	–1.25	ns	–1.65	0.37
Somatization (SOM)	2.17	0.74	2.93	< 0.001	0.72	3.63
Obsessive-compulsive (OC)	7.80	0.69	11.33	< 0.001	6.45	9.15
SOM*OC	–2.72	0.77	–3.54	< 0.001	–4.23	–1.21

**SE, Standard Error; 95% CI, 95% Confidence Interval; unstandardized betas.**

**FIGURE 1:**
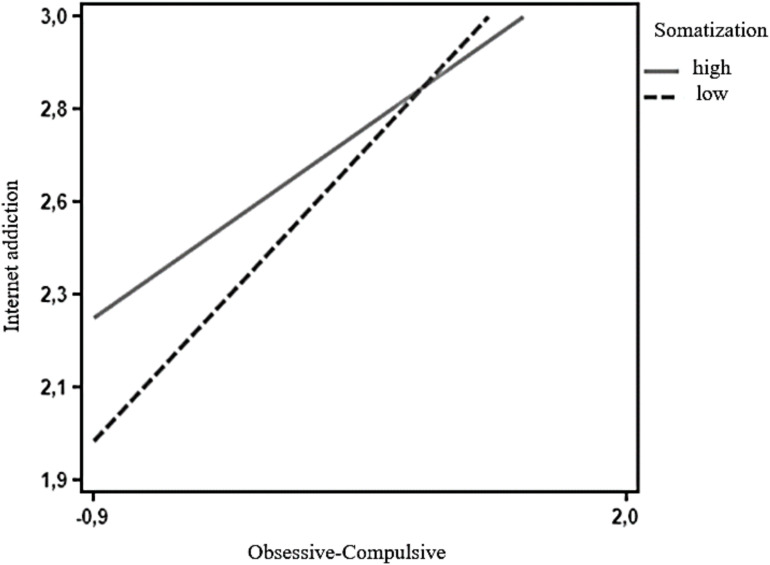
Interaction term for two levels of somatization: low (−1 SD) and high (+1 SD). Using the Johnson-Neyman technique (Bauer and Curran, 2005), we identified the region where the effect of somatization symptoms on the relationship between OC and IA ceased to be statistically significant. Application of the Johnson-Neyman technique gave cutoff scores for somatization symptoms of below 1.81 and above 1.63.

Simple slopes analyses revealed that when somatization symptoms were low (−1 SD), there was a statistically significant effect of obsessive-compulsive symptoms on increased IA (β = 9.48, *SE* = 0.88, *t* = 10.80, *p* < 0.01). Furthermore, also when somatization symptoms were high (+1 SD), there was a significant effect of obsessive-compulsive symptoms on IA (β = 6.13, *SE* = 0.79, *t* = 7.83, *p* < 0.001). Simple slopes analyses (Figure 1) revealed that when somatization symptoms were low.

## Discussion

The aim of this study was to increase current knowledge about the relationship between somatization symptoms, obsessive-compulsive symptoms, and IA in adolescents. Specifically, we hypothesized that the relationship between obsessive-compulsive symptoms and IA would be stronger at higher levels of somatization symptoms.

First, findings from our study suggest that obsessive-compulsive symptoms are associated with IA. These results are in line with prior research, which found that high levels of obsessive-compulsive symptoms are linked to higher IA risk (Jang et al., 2008; Dong et al., 2011; Ko et al., 2012). Furthermore, IA has typically been described as a secondary condition resulting from various primary disorders, although findings in young adult samples have suggested that, within a range of psychopathologies, only obsessive-compulsive symptoms preceded IA (Dong et al., 2011; Ko et al., 2012). The obsessive-compulsive symptoms observed in association with IA are similar to those of OCD, so much so that many researchers define IA as compulsive computer use (Kuss et al., 2014). However, the obsessive-compulsive symptoms of OCD have been described as more ego-dystonic than those of IA (Shapira et al., 2000). In general, the obsessive-compulsive symptoms of IA stem from recurring or persistent positive or negative thoughts (obsessions) that motivate the individual to implement behaviors (compulsions) intended to allow him/her to experience the hedonic satisfaction deriving from obtaining a positive reinforcement (Robbins and Clark, 2015), or to reduce the distress typically associated with craving and abstinence states. IA may thus serve as a strategy for relieving pre-existing obsessive-compulsive psychopathology, a mechanism that, in turn, could actually reinforce the symptoms (Ko et al., 2012). Similarly, this association could be further reinforced by underlying mechanisms shared by OC and IA behaviors (Ko et al., 2012). Repetitive behavioral manifestations aimed at achieving immediate gratification or de-escalating the distress triggered by obsessive thoughts in order to improve one’s feelings are typical of addictions and compulsive behaviors (Robbins and Clark, 2015). In the present study, the main effect of somatization symptoms on IA was in line with the findings of previous research (Yang et al., 2005; Yen et al., 2008; Alavi et al., 2011; Yao et al., 2013). Somatization is conceptualized as a process that leads to translation of psycho-emotional distress into bodily discomfort (Nakkas et al., 2019). Subjects with somatization disorders requiring inpatient treatment manifest deficits in both emotional awareness and Theory of Mind functioning. These deficits may underlie the phenomenon of somatization (Subic-Wrana et al., 2010).

As regards our moderation hypothesis, we found that the relationship between obsessive-compulsive symptoms and IA was greatest at higher levels of somatization symptoms. Our results showed that in adolescents with higher somatization (+1 SD), the relationship between obsessive-compulsive symptoms and IA was stronger. To our knowledge, this is the first study that has investigated this relationship. Our results are in line with the triadic theory of addiction (Noël et al., 2013), where somatization, as a major expression of the enteroceptive system, could hinder the management of normal emotional distress through problem-focused coping strategies based on reflexive system mentalization skills. This apparent partial impairment of the reflexive system’s capacity to regulate emotional distress could therefore lead adolescents to adopt emotion-focused coping strategies, such as ones related to implementation of the same obsessive-compulsive behaviors promoted by the impulsive system. Somatization could therefore impair the mentalization skills used by the reflexive system to inhibit compulsive behaviors driven by the impulsive system, predisposing the adolescent to develop IA. This could explain why obsessive-compulsive symptoms are often found in the literature as prodromes of IA development (Dong et al., 2011; Ko et al., 2012), as well as why IA has typically been described as a secondary disorder resulting from a primary one, like obsessive-compulsive symptomatology (Dong et al., 2011; Ko et al., 2012), a relationship that is confirmed in our study.

Our analyses were performed controlling for gender, age, grade, and school. Specifically, a significant gender difference emerged, as showed in previous studies (Cao et al., 2011; Barke et al., 2012; Kuss et al., 2013). As showed in a study by Feng et al. (2019), we have found a significant grade difference.

The present study has several limitations. First, the cross-sectional design used does not allow the identification of causal relationships among variables. We cannot definitively conclude that obsessive-compulsive symptoms cause IA and that this relationship depends on levels of somatization. Future studies should consider longitudinal data to overcome the cross-sectional limitations. A second, potential, limitation concerns the reliance on self-reported data, which might have caused common method bias. However, we ran the Harman’s single factor test, which suggested that common method bias did not affect the results of this study. A third limitation concerns mentalization ability. Good mentalization could be protective against somatization, but we did not measure it. Future research could explore this aspect through specific questionnaires.

## Conclusion

Adolescence is an important period of physical and psychological development. From a clinical perspective, the results of this study show that somatization is an important moderation factor in adolescence. The incapacity to use coping strategies and mentalization strategies to counter negative emotions could increase the somatization effect. In adolescents, obsessive-compulsive symptoms can be moderated by somatization. In this period of development, it is very important to pay attention to bodily signals, as they can mask psychological problems. Obsessive-compulsive symptoms can be very invalidating, and they can be exacerbated by somatization. Teenagers seeking a coping response in technological devices are at considerable risk of developing pathological use of these devices.

In conclusion, somatization is an important aspect to consider when dealing with adolescent patients. It could be a moderation factor capable of exacerbating obsessive-compulsive symptoms or IA. This particular aspect needs more studies in the future.

## Data Availability Statement

The raw data supporting the conclusions of this article will be made available by the authors, without undue reservation, to any qualified researcher.

## Ethics Statement

The studies involving human participants were reviewed and approved by the CARU-Comitato di Approvazione per la Ricerca sull’Uomo, Università di Verona. Written informed consent to participate in this study was provided by the participants’ legal guardian/next of kin.

## Author Contributions

FL, AR, and LZ were responsible for the study concept and design. SC, FC, and RM contributed to the data acquisition. IP assisted with the data analysis and interpretation of findings. AF, LZ, IP, and AC drafted the manuscript. All authors critically reviewed the content and approved the final version of the manuscript for publication.

## Conflict of Interest

The authors declare that the research was conducted in the absence of any commercial or financial relationships that could be construed as a potential conflict of interest.
